# Do Rubber Floor Mats Prevent Lameness in Gestating Sows Housed in Large Groups? A Field Experiment on Three Commercial Farms in France

**DOI:** 10.3390/ani11113160

**Published:** 2021-11-05

**Authors:** Adeline Huneau-Salaün, Stéphanie Bougeard, Loïc Balaine, Florent Eono, Éric Eveno, Maxime Guillermic, Rodolphe Thomas, Nicolas Rose, Françoise Pol

**Affiliations:** 1 Agence Nationale de Sécurité Sanitaire de L’alimentation, de L’environnement et du Travail (Anses), B.P. 53, 22440 Ploufragan, France; stephanie.bougeard@anses.fr (S.B.); loic.balaine@anses.fr (L.B.); florent.eono@anses.fr (F.E.); eric.eveno@anses.fr (É.E.); rodolphe.thomas@anses.fr (R.T.); nicolas.rose@anses.fr (N.R.); francoise.pol@oniris-nantes.fr (F.P.); 2 Cooperl Arc Atlantique, 7 Rue de la Jeannaie, 22400 Lamballe, France; maxime.guillermic@cooperl.com

**Keywords:** sow, lameness, soil lining

## Abstract

**Simple Summary:**

In the European Union, gestating sows are housed in groups in pens that often have slatted floors. Slatted floor can cause injuries to the animals’ legs and hooves, leading to potentially painful lameness. The purpose of our study was to verify if the installation of rubber mats on their floors could limit the occurrence of injuries and lameness in gestating sows. Mats were installed on three commercial farms for use over two consecutive sow pregnancies. The mats limited the occurrence of inflammations around the sows’ leg joints but had no impact on the occurrence of lameness or leg and hoof injuries. The benefit of mats would be greater if they were available throughout the sows’ lives and not just during the gestation period. However, the mat design tested was unsatisfactory because manure did not drain well off the mat and sows were dirtied quickly.

**Abstract:**

Lameness and foot disorders are major health and welfare issues in intensive swine production systems. They are exacerbated when sows are housed in large groups on slatted concrete floors during gestation. Our study aimed to assess the effect of rubber mats in the lying area of the gestation pen on lameness and leg health in gestating sows housed in large pens in commercial conditions. The study was conducted on three commercial farms over two successive gestations. A total of 582 Large White × Landrace sows, housed in 10 static groups, were enrolled: 5 groups in pens with rubber mats and 5 groups on slatted concrete floors. Lameness, bursitis, leg injuries, claw growth defects and claw lesions were measured at the beginning, middle and end of each gestation period. The rubber mats decreased the risk of suffering from bursitis, but had no effect on the risk of lameness, leg injuries, claw growth defects or claw lesions. Sows housed on rubber mats were heavily soiled compared with those on slatted concrete floors because the mats were not perforated for slurry evacuation. Locomotion disorders and foot lesions remained prevalent despite the rubber mats in the lying area of the gestation pens, but adding rubber mats in service rooms and farrowing crates may produce better results.

## 1. Introduction

Lameness in sows is a major health problem in intensive swine production systems. Studies across Europe have revealed that the prevalence of lameness on farms ranges from 9 to 29% [1,2,3,4,5,6]. This painful disorder can have multiple infectious or non-infectious aetiologies, including foot and leg injuries [7]. Pain due to foot and leg injuries is one of the major welfare risks identified for gestating sows [8]. Lameness is also an economic issue, because lame sows are less productive [9,10] and culled earlier than non-lame sows [3,11,12]. Lameness is also one of the major reasons for on-farm deaths or euthanasia [13,14].

Housing on slatted concrete floors, which is the main type of flooring for gestating sows in France [15], has been reported as a main cause of lameness [1,2,4,16,17,18]. Concrete floors can lead to excessive abrasion of claws. Wet concrete floors are also slippery and may cause leg injuries. Claws can get caught between the slats and be pulled off. On the contrary, housing sows on soft floors may reduce leg disorders. As an example, several studies have demonstrated the benefit of straw bedding compared with slatted concrete floors on lameness and foot health of sows and gilts [1,2,4]. However, straw litter, even on just part of the floor, is not compatible with slatted floors and liquid-manure handling systems. Therefore, rubber mats on slatted floors in the lying areas may be a solution to improve floor softness without impairing slurry evacuation by scraping. Mats increase the contact area between the animal’s body and the floor and limit push forces on body parts in contact with the floor [19], thereby improving the comfort of animals lying down on the floor. Using a preference test, Parsons et al. [20] demonstrated that lame sows preferred rubber mats to concrete floors.

Positive effects of rubber mats on the reduction of locomotor disorders have already been described for growing pigs [21,22]. In farrowing crates, rubber mats decrease sow shoulder ulcers [23,24,25,26,27,28]. In service crates, they limit claw lesions [29]. In gestation pens, rubber mats improve leg health of sows housed in small groups (four to eight animals), but show inconsistent results on lameness [30,31,32]. In larger groups of gestating sows, rubber matting has showed positive effects on overall leg health [33] and on lying comfort [34]. However, the effect on lameness has not been assessed [34] or has been assessed on small groups (20–25 sows) in experimental conditions only [33]. The assessment of the impact of rubber mats (in the lying area) on lameness in large groups of gestating sows is of importance because group housing—especially large group housing—has been identified as a risk factor for lameness [1]. Given that housing gestating sows in groups is mandatory in the European Union [35], rubber mats may reduce the risk of lameness associated with this housing system.

Our study aimed to assess the effect of rubber floor mats installed in the lying area of large gestation pens on lameness and leg health of gestating sows housed in commercial conditions. The sows were followed over two successive gestations. Several parameters on claw lesions, body condition and performance were also monitored.

## 2. Materials and Methods

The experimental procedure was designed at the officially approved organization for animal experimentation ANSES (official number D22-745-1). The experiment lasted from January 2016 to August 2018 and was then conducted during all seasons and under different thermal conditions, as recommended by EFSA [36].

### 2.1. Study Population

The study was conducted on three commercial farrow-to-finish farms located in Brittany, western France. These farms previously encountered lameness issues in sows. The prevalence of lameness in the three farms was 22% (confidence interval of 95% [18,19,20,21,22,23,24,25,26]) at the beginning of the study. Two of them (Farms A and B) had 250 sows and the third (Farm C) had 700 sows. The longitudinal study monitored Large White × Landrace sows housed in 10 static groups, over 2 successive gestations, from 6 weeks before the first service (Week 0) to 1 week before the second farrowing (W33). During the gestation period, the groups were made up of sows at the same stage of gestation, but with different parities: 4 groups of 50 sows on Farm A, 4 groups of 45 sows on Farm B and 2 groups of 95 sows on Farm C. During service, from 1 week after weaning to 4 weeks after insemination, sows were housed in individual stalls floored with concrete slats. During gestation, sows were housed in group pens as described below in the experimental design. During the farrowing and suckling periods, sows were housed in individual farrowing crates with standard slatted metal flooring. Piglets were weaned at 28 days old.

All sows present within the groups were observed. Some sows were removed from the groups and from the experiment due to reproductive failure or culling. Conversely, nulliparous sows were added to the groups at the second gestation cycle. Consequently, group composition changed over cycles. Over the 58 visits carried out on the farms, 582 sows were observed at least once: 220 sows on Farm A, 186 on Farm B and 176 on Farm C. Due to feasibility constraints, not all sows could be observed during each visit and the number of observations varied among sows. When the study started, 17% of the sows were primiparous and 11% had a parity of greater than five.

### 2.2. Experimental Design

**Figure 1:**
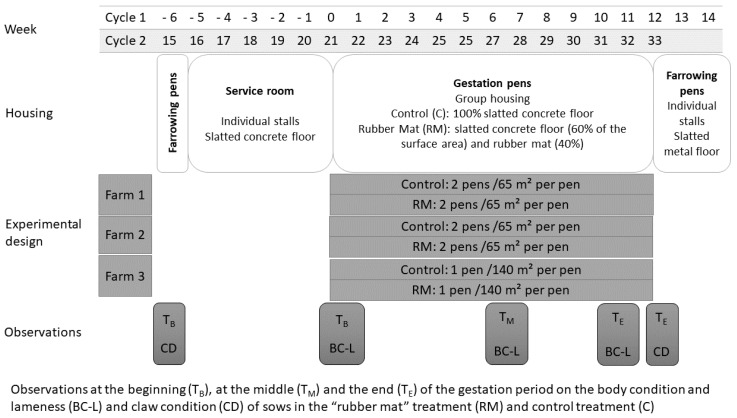
Experimental protocol.

Rubber mats were tested during the two gestation periods, from W0 to W12 and from W21 to W33 (Figure 1). Monitored gestating sows were housed in 10 group pens on the 3 farms: 4 on Farm A (4 m × 65 m), 4 on Farm B (4 m × 65 m) and 2 on Farm C (2 m × 140 m). In half of the group pens, evenly distributed across farms, the lying area was covered with rubber mats. This area represented 40% of the floor in the group pens. The rest of the area remained a fully slatted concrete floor with a permeability less than 15% in accord with the European regulation [35]. The floor of the other pens, considered control pens, was fully slatted concrete. The interval between two batches of sows was three weeks. During the first monitored gestation period, groups of sows were allocated to rubber mat or control treatments alternately every 3 weeks. Thus, five groups of sows were housed in pens partially lined with mats (“rubber mat”–RM treatment) and five groups were housed in pens with fully slatted concrete floors (“control”–C treatment). Each group of sows was allocated to a pen and stayed on the same type of floor during the two gestation periods. The animals were housed at a density defined by Directive 2008/120 [35] and fed using an electronic feeder (liquid feed for farms B and C and solid feed for farm A). Water was freely available from nipple drinkers.

The rubber mats (BioretAgri, Nort-sur-Erdre, France) were made of a foam underlayer for suppleness, and a rubber upper layer (thickness: 10 mm) (Supplementary file 1). The rubber layer was grooved to provide a non-slip surface and contained woven thread to improve durability. Mats were attached in pens’ laying boxes using baseboards nailed to the partitions and threshold bars nailed to the slatted floor in the open part of the box. The mat was not perforated and it had a slight slope (3%) to favour slurry runoff.

### 2.3. Measures and Data Collection

The sows were observed in gestation and farrowing pens and during transfers between rooms, the most convenient places and times for observations. Sow lameness was scored during the transfer between the service room and the gestation pen at W0 and W21 (Tb, beginning of gestation time), inside the gestation pen at W6 and W27 (Tm, mid-gestation) and during transfer between the gestation pen and the farrowing pen at W12 and W33 (Te, end of gestation) (Figure 1). Transfer corridors had a concrete floor. The sows were observed while they walked a distance of at least 15 m, except very lame sows, which were not forced to walk. Lameness was scored according to a four-category table based on the Welfare Quality protocol [37]. Non-lame sows (0) were distinguished from slightly lame sows (1) who had difficulties walking but were still using all theirs legs. A score of two corresponded to animals with a severe lameness; they put a minimum weight on the affected limb; a score of three was given to animals unable to walk. For statistical analysis, scores of two and three were aggregated in the “severe” class. On the same occasions than lameness observations, wounds on the legs and manure on the body were measured according to the Welfare Quality three-category scales, scored on both sides of the sows. For the wound score, the scale varied from zero (no visible injury or up to four lesions) to two (more than 15 visible lesions). Scores for manure on the body ranged from zero (up to 10% of the body surface soiled) to two (more than 30% of the body surface soiled). Scores of two and three were aggregated in the “very soiled” class for statistical modelling. Bursitis was counted on all four legs. Body condition was assessed using a three-category score derived from the Welfare Quality (zero—thin, one—normal condition, two—fat).

Claw lesions were visually scored on the hind legs when the sows were lying in farrowing pens on four occasions (W-6, W12, W15 and W33) for the convenience of the observations of the four legs. The three-category scoring system for claw lesions was based on the lesion scoring table proposed by Enokida et al. [38]: zero (no lesions), one–two (superficial cracks and/or overgrowth), and three (deep cracks or wrenching). The heel, the junction between the heel and the sole, the white line (junction between the sole and the wall), the wall, the growth of major claws and the growth and integrity of dewclaws were scored. Scores for the heel, junction, white line and wall of the two hind claws were summed to obtain an erosion score (from 0 to 12). An erosion score of zero or one was considered “no erosion”, a score between three and six “mild erosion” and a score over six “severe erosion”. Scores for the growth of major claws and dewclaws were summed to obtain a growth score for each sow (over 24).

Measurements were taken by six trained assessors. Training sessions of observations were carried out for ensuring inter-observer agreement at the beginning of the study. To control observation bias, each assessor monitored the same sows, equally distributed in RM and C groups within each farm, from the beginning to the end of the experiment. Reproductive performance, in terms of number of piglets born, number of piglets born alive and number of weaned piglets, were recorded for all sows over the two gestation periods.

### 2.4. Statistics

Scores obtained at the beginning of the Cycle 1, before affecting sows to treatments, were considered as baseline for ulterior comparisons. Lameness, bursitis, claw defects and body condition are shown in terms of frequencies of observation and of numbers of sows affected at least once over the study period. Distributions of erosion score and claw growth score are given. All statistical analysis were performed in R version 3.6.2 [39]. Factors considered for statistical analysis were Treatment (C and RM), period of gestation (Tb, Tm for lameness and bursitis and Te), cycle of gestation (Cycle 1, Cycle 2) and parity of the sow when enrolled (first gestation, parity 2 to 5 and parity >5). These four factors were introduced as fixed effects in generalized linear mixed models (GLMM). A random component was introduced in the models accounting for the effects associated with a sow, within a pen, within a farm (nested effects). The Treatment × Cycle interaction was tested for all models and removed if non-significant (*p* > 0.05). Risks of suffering from bursitis, leg injuries, scratches, and abnormal body condition (thin, fat) were modelled using a mixed-effect logistic regression model (binary response as affected vs. non-affected); the function glmer from the lme4 package [40] was used. Other measures related to lameness, claw defects and body cleanliness were categorized on a three-score scale (absence–mild–severe) according to score frequencies. The risks of suffering from mild and severe conditions were modelled using an ordinal regression based on a cumulative link mixed model (function clmm from the ordinal package [41]). Results from the GLMM models are reported as odds ratios adjusted on the fixed effects with their confidence intervals at 95%. A factor with an odds ratio higher than 1 (i.e., the lower limit of the OR’s confident interval at 95% is higher than 1) increases the risk of suffering from the studied condition. In contrast, a factor with an odds ratio lower than one (upper limit of the OR’s Confident Interval at 95% is lower than 1), can be considered as a protective factor. Odds ratios and their confidence intervals are shown in figures and the complete results of the models are provided (Supplementary file 2). Reproductive performance traits were analysed using ANOVA mixed models with Treatment, Cycle and Parity as fixed effects and Pen, nested in Farm, as a random effect (lmer function from lme4 package). Results are shown as least square means with standard error. Post-hoc comparisons of means were performed using the Tukey correction (package emmeans [42]) for multiple comparison adjustments.

## 3. Results

At the beginning of the study, the prevalence of lameness was similar within treatments: 23% ([17,18,19,20,21,22,23,24,25,26,27,28]) in treatment RM vs. 21% ([16,17,18,19,20,21,22,23,24,25,26]) in treatment C (*p* = 0.33). No Treatment*Cycle interaction (*p* < 0.05) was observed for any of the parameters under study, suggesting that the effect of rubber mat was constant over the two observed gestation periods.

### 3.1. Lameness

**Figure 2:**
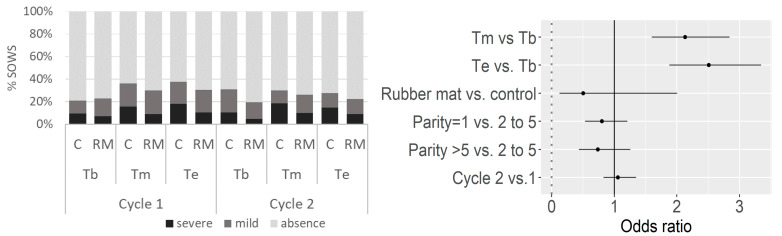
Frequencies of observed lameness and estimated odds ratios (with 95% confidence intervals) according to the floor type and gestation cycle, from the beginning (Tb) to the middle (Tm) and end (Te) of the gestation period. RM, rubber mat; C, control.

Lameness was reported for 28% of the observations (663/2361), from a moderate state (17% of the observations) to a severe state on 39 occasions (2% of the observations, Figure 2). From the 495 sows observed for lameness scoring (no data available for 87 sows due to feasibility constraints), 256 were lame at least once (52%) and 7 sows exhibited severe lameness. The risk of suffering from mild or severe lameness increased during the gestation period: the odds ratio (OR) was 2.1 (CI 95% [1.6–2.8]) when comparing Tm with Tb (*p* < 0.001) and was 2.5 ([1.9–3.3], *p* < 0.001) when comparing Te with Tb (Figure 2). No effect of RM treatment was observed on the risk of suffering from lameness (RM vs. C, OR = 0.50 [0.13–2.0], *p* = 0.33).

### 3.2. Leg Condition

**Figure 3:**
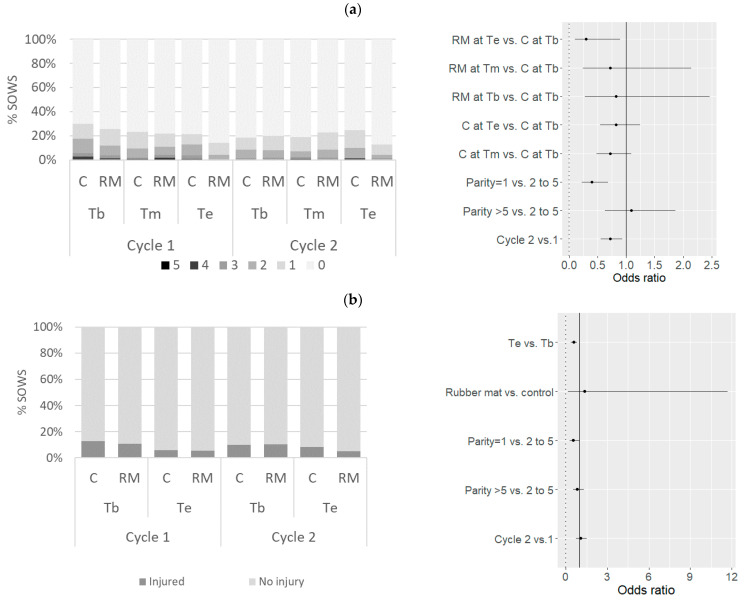
Frequencies of observed (**a**) bursitis and (**b**) leg injuries and estimated odds ratios (with 95% confidence intervals) according to the floor type and gestation cycle, from the beginning (Tb) to the middle (Tm) and end (Te) of the gestation period. RM, rubber mat; C, control.

Bursitis was noted on 21% of the observations, affecting 226 sows at least once (39% of the sows, Figure 3). Up to five bursitis were observed on a sow on three occasions, but 55% of the observations corresponded to one bursitis affection only. The risk of being affected by bursitis (at least one bursitis) was significantly lower at the end of the gestation period for the sows on rubber mats only: in the RM treatment, the OR associated with Te vs. Tb was 0.42 [0.20–0.60] (*p* = 0.03). On the contrary, the frequency of bursitis was unchanged over the gestation period for sows in the C treatment (OR associated with Te vs. Tb = 0.8 [0.55–1.21], *p* = 0.35). Primiparous sows were less frequently affected with bursitis (OR = 0.39 [0.22–0.68], *p* = 0.001) than older sows. The risk of suffering from bursitis turned out to be lower during the second gestation cycle than the first one (OR = 0.71 [0.55–0.93], *p* = 0.01)). Presence of leg injuries was reported for 142 observations out of 1650 (9%); 115 sows (20%) exhibited wounds on least at one visit. The probability that a sow suffers from leg injury was lower at the end of the gestation period compared with the beginning (OR = 0.55 [0.38–0.80], *p* = 0.002), but was not influenced by the rubber matting (OR = 1.4 [0.16–11.7], *p* = 0.77) in comparison with concrete floor.

### 3.3. Claw Condition

**Figure 4:**
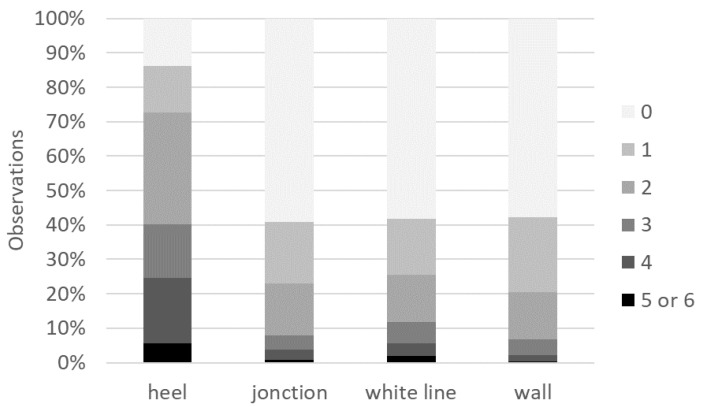
Frequencies of claw lesion scores (from 0—no lesions to 6—severe lesions) according to the area of the hind claw.

**Figure 5:**
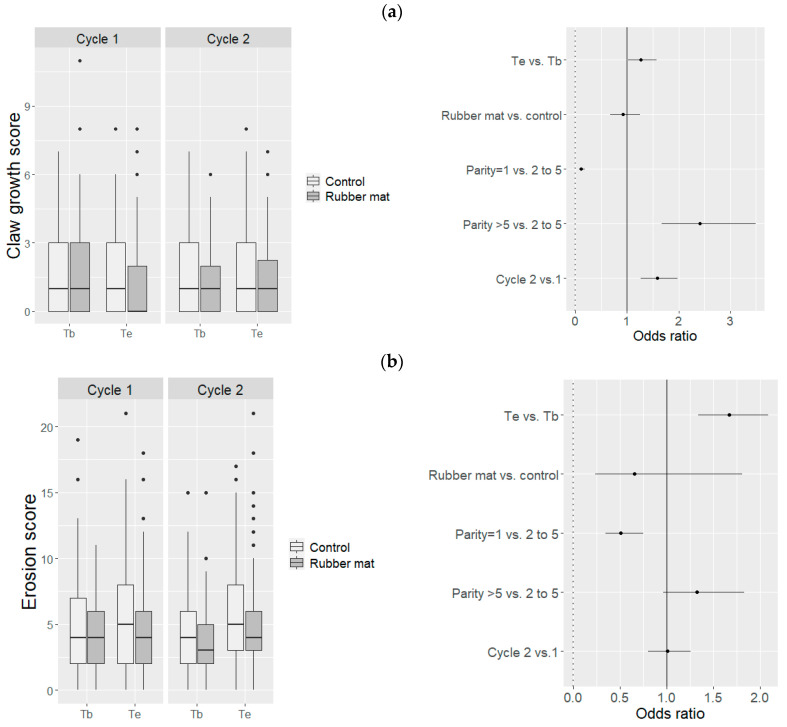
Distribution of (**a**) claw growth-defect scores, (**b**) claw-erosion scores and estimated odds ratios (with 95% confidence intervals) related to mild or severe forms of those conditions according to the floor type and gestation cycle, at the beginning (Tb) and the end (Te) of the gestation period.

Dewclaws exhibited lesions more frequently (54% of the observations) than major claws (12%). Out of 576 sows, 174 (30%) showed a claw growth defect score (sum of scores on dewclaws and major claws) higher than 3 out of 6 (severe) at least upon one observation; only 159 sows (28%) had no claw defects (score 0) over the whole study period. Heels were more heavily worn than the other parts of the hind claws (Figure 4). No erosion (score 0 or 1) was observed for 11% of the observations, mild erosion (score [2,3,4,5,6]) for 66% of the observations and severe erosion (score over 6) for 23% of the observations. Overall, 224 sows (out of 576, 39%) had a severe erosion score on at least one occasion, whereas 27 sows (5%) had a “no erosion” score over the whole study period. The presence of a rubber mat had no effect on the claw growth-defect score (OR = 0.92 [0.67–1.25], *p* = 0.59) or on erosion score (OR = 0.65 [0.24–1.81], *p* = 0.41) (Figure 5). The probability of observing claw growth defects was greater (OR = 1.6 [1.3–2.0], *p* < 0.001) during the second gestation cycle than the first one. The risk of “mild” or “severe” erosion of claws was higher at the end of the gestation period than at the beginning (OR = 1.7 ([1.4–2.1], *p* < 0.001). Claws of sows with parity >5 at the beginning of the study were more worn (OR =2.4 [1.7–3.5], *p* < 0.001) than those of younger sows.

### 3.4. Body Condition

**Figure 6:**
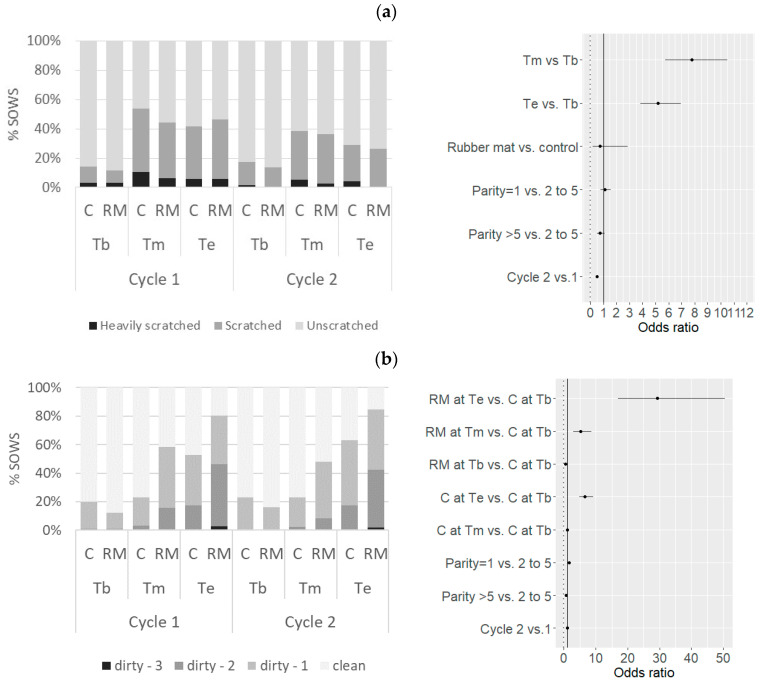

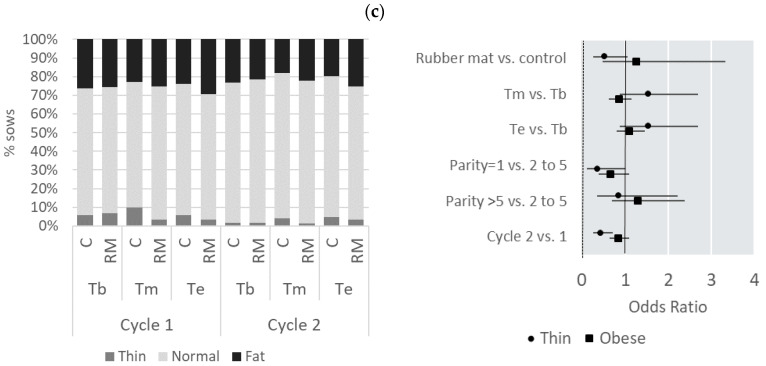
Frequencies of (**a**) body scratches, (**b**) body-cleanliness scores, (**c**) body conditions and estimated odds ratios (with 95% confidence intervals) related to mild or severe forms of these conditions according to the floor type and gestation cycle, from the beginning (Tb) to the end (Te) of the gestation period. RM, rubber mat; C, control.

About two-thirds of the sows were scratched (756/2361 observations, 68%), including 4% heavily scratched sows. Two-thirds of the sows were scratched on at least one occasion (68%, 336/495). The risk of being scratched increased during the gestation period (OR associated with Te vs. Tb = 7.7 [5.8–10.5], *p* < 0.001) (Figure 6). No effect of the RM treatment was observed (OR associated with RM vs. C = 0.73 [0.19–2.9], *p* = 0.66). Scratches were less frequent during the second cycle than the first one (OR = 0.52 [0.42–0.66], *p* < 0.001). Sows were judged as clean in 58% of the observations (1377/2360 observations) whereas scores for lightly soiled (1), soiled (2) and heavily soiled (3) sows accounted for 29%, 12% and 0.4% of the observations, respectively. State-of-cleanliness strongly varied over the gestation period and among treatments, as shown by the significant Time*Treatment interaction. Sows in the RM treatment became soiled more quickly and more heavily than those in the C treatment: at Tm, the OR associated with RM vs. C was 4.3 [2.5–7.6] (*p* < 0.001) and at Te, the OR associated with RM vs. C was 3.5 [2.0–6.3] (*p* < 0.001). The older sows (parity >5 at the beginning of the study) were less at risk of being soiled than the other sows (OR = 0.70 [0.49–0.99] in comparison with sows with parity 2–5, *p* = 0.05). Conversely, primiparous sows at the end of the gestation period were dirtier than the other sows (OR = 1.6 [1.2–2.2], *p* < 0.001). Thin sows were reported for 108 observations over 2353 (5%) and 15% of the sows (73/495) were judged as thin at least once. Forty-three percent of the sows (213/495) were judged as fat at least at one visit, accounting for 24% of the observations (561/2353). Frequencies of abnormal body condition (thin or fat) were not affected by the type of floor. The risk of thinness (in comparison with normal condition) was lower during the second cycle of gestation (OR = 0.43 [0.26–0.72], *p* < 0.001).

### 3.5. Reproductive Performance

The numbers of born piglets, viable piglets at birth and weaned piglets per sow were not affected by the floor lining in the gestation pen (Supplementary file 2). The number of weaned piglets per sow was higher for Cycle 1 (12.6 [11.5–13.7], *p* < 0.001) than Cycle 2 (12.0 [10.4–13.7]) and for primiparous sows (12.8 [12.4–13.2]) or parity 2 to 5 (12.4 [12.1–12.7]) in comparison with older sows (11.7 [11.3–12.1], *p* < 0.001).

## 4. Discussion

The aim of this study was to assess the impact of a rubber mat floor lining on lameness and foot health of gestating sows housed in large groups in commercial conditions. Adding a rubber mat floor lining in the gestation pens protected sows from bursitis development during the gestation period. However, it did not improve lameness, claw state or body integrity. Additionally, the mats, which were not perforated, impaired slurry evacuation. Sows on rubber mats became more quickly and more heavily soiled than sows kept on fully slatted floor. Previous studies reported results on the impact of rubber mats on foot health and on the risk of lameness in sows [21,23,27,29,30,32,33]. However, our study provides new results obtained on pregnant sows housed in large groups and under real production conditions, which have never been studied before.

No protective effect of the rubber mats was observed for lameness, claw erosion, claw defects, leg injuries, body condition or reproductive performance. In previous studies, rubber matting has shown a beneficial effect on locomotor disorders in experimental conditions, particularly during periods when sows were housed in crates with rubber mats [30,31,33]. In commercial conditions, the beneficial effects of rubber mats would possibly have been observed if the mats had been installed over the entire area of the pen. Prior to this study, we tested groups pens fully lined with perforated rubber floor mats during a pilot study. However, the perforations in the mats did not line up with those of the slatted floor and problems of slurry evacuation occurred. It was impossible to produce perforated mats that perfectly matched with the slats of the floor because the slatted floors were different in the three studied farms. Slatted floors directly covered with a layer of soft material can be considered to cover the whole surface of the gestation pen but this solution implies changeing the slatted floor. Better results may be possible if the sows were to spend more time on a more comfortable floor, i.e., in their service rooms and farrowing pens. Sows were kept in gestation pens for only half of the production cycle (12 out of 21 weeks). Nevertheless, poor locomotion scores remained high on both floor types, as already described in previous studies [31,43]. The prevalence of mild and severe lameness was the highest at the end of the gestation period, as in previous studies [31,43]. Conversely, lameness decreased between the two gestation periods, probably because the sows were housed in individual stalls during farrowing and service and could no longer walk [43].

Rubber mats in the resting areas significantly decreased the risk of suffering from bursitis. Bursitis is an inflammation of the bursa surrounding a joint. Inflammation is caused by repeated pressure or friction at the joint and can be a source of pain [44]. Bursitis is also considered a risk factor for lameness [45]. Bursitis count is one of the control points in the Welfare Quality protocol for assessing floor comfort. In our study, lying on a softer floor reduced the number of potentially painful bursitis-affected joints and was more favorable to the welfare of the animals, as demonstrated previously [30].

Occurrence of claw growth defects and state of claw erosion were not influenced by the presence of rubber mats. The frequency of leg injury was also unaffected by the type of floor. Previous experimental studies reported mixed effects of rubber mats on the foot health of gestating sows [32,46]. In a study focused on the beginning of gestation, when sows are mixed in a pen, Elmore et al. [32] observed a reduction of lesions on the bodies of sows housed on rubber mats in comparison with sows housed on concrete floor. However, no difference was observed for leg injuries. Similarly, Jais et al. [46] reported no effect of rubber mats on leg injury of sows followed during two years (up to five gestations), but claw-horn erosion was less severe for sows on rubber mats. In this study, rubber mats did not improve claw state in terms of horn fracture and growth defects. We observed no significant effect of rubber mats on the severity of claw erosion and on the occurrence of claw defects in our study, but the study period (two gestation cycles) may not be long enough to observe the long-term effects of rubber matting on claw health. Erosion and claw defects increase when sows get older, as shown in our study for sows with a parity greater than five.

Regarding body condition, the risk of being scratched was as high in sows on rubber mats as in sows on concrete slatted floor. Most of the scratches observed were due to fights when sows were grouped in gestation pens. No influence of the type of floor was observed on sow body condition, in line with results obtained on lactating sows provided with rubber mats [27]. In contrast, body cleanliness was poor for sows on rubber mats at the end of the gestation period. The gentle slope on the rubber mat surfaces was not sufficient to ensure optimal slurry evacuation. In fact, the mats were so dirty that we could not assess their state of wear during the gestation periods. However, body cleanliness is an important factor for welfare, in particular for the thermal comfort of the sows (Welfare Quality, 2009). Perforated rubber mats are highly recommended to maintain the body cleanliness of sows [30,31]. In addition, several mats were replaced between the two gestation cycles because the sows had chewed them—in particular, on one farm where the sows were not provided with enrichment material during the first gestation, despite the experimental design and the mandatory practices stipulated in Directive 2008/120. Foraging and chewing are important activities in normal pig behavior. In absence of chewable material, this behavior may be redirected to material present in the pen, such as the rubber mats. Therefore, providing enrichment material, even sub-optimal material such as metal chains, is needed to improve sow welfare and prevent the excessive wear of the mats.

Reproductive performance traits were similar between sows on rubber mats and sows on slatted concrete floors, in terms of litter size, number of piglets born alive and number of weaned piglets per sow. In the Lagoda et al. study [47], gilts housed on rubber mats experienced less intense aggression when grouped in the gestation pens than the gilts housed on slatted concrete floors. As a consequence, the number of dead-born piglets was lower for the sows on the rubber mats at the second farrowing. This may be due to the less stressful environment for those sows. However, the overall productivity of the sows was not affected by the type of floor lining, as in the present study. In fact, sow lameness seldom affects reproductive performance and piglet health [2]. As rubber mats in the laying area did not affect the occurrence of lameness in sows in the present study, it was unlikely to observe an increase in reproductive performance and in piglet performance. Given that improvement in reproductive performance traits is expected to be low in sows on rubber mats, longitudinal cohort studies on a small number of farms may not be the most appropriate study design from which to draw conclusions on these parameters. Large surveys based on performance data are likely to be more statistically powerful for that purpose. The number of weaned piglets was higher for the second cycle of gestation in comparison with the first gestation cycle. The first gestation cycle was from June to October, whereas the second gestation period was from December to March in the three studied farms. Sow fertility and prolificacy are known to decrease during summer [47]. Hot temperatures may have negatively affected reproductive performance during the first gestation cycle. Other factors might decrease reproductive performance, like the parity rank. Primiparous and aged sows usually exhibit lower reproductive performance than females with parity ranks between two and five [47], as observed in the present study.

## 5. Conclusions

Housing pregnant sows on floors partially lined with rubber mats instead of a fully slatted floor had some positive effects, such as reducing the occurrence of bursitis. However, the overall effect of the rubber mat lining was limited on lameness, foot and claw health and therefore on sow welfare. Locomotion disorders and foot lesions remained prevalent despite the installation of rubber mats. Better results may be expected with the use of rubber mats in service rooms and farrowing crates also, i.e., during the whole servicing period of the sows. The interest of rubber mats should therefore be tested in a more global approach, covering the entire servicing period and integrating other parameters such as the provision of adequate chewing material. The design of rubber mats also needs to be improved (more efficient perforations for slurry evacuation, improved resistance to chewing) to develop their use in commercial pig farms.

## Data Availability

The data presented in this study are available on request from the corresponding author. The data are not publicly available due to privacy.

## Supplementary Materials

The following are available online at https://www.mdpi.com/article/10.3390/ani11113160/s1, Supplementary file 1: Rubber mat’s conception, Supplementary file 2: Results of the GLM models.
