# Supplementary material for: Do Rubber Floor Mats Prevent Lameness in Gestating Sows Housed in Large Groups? A Field Experiment on Three Commercial Farms in France
Source: Animals (Basel). 2021 Nov 5;11(11):3160. doi: 10.3390/ani11113160 (PMC8614336; doi:10.3390/ani11113160)
Supplement: Supplementary file 1 [file animals-11-03160-s001.zip › Supplementary_material_S1_v2.pdf]

Do Rubber Floor Mats Prevent Lameness in Gestating Sows Housed in Large Groups? A Field Experiment on Three Commercial Farms in France

Supplementary material S1: Rubber mat's conception

a)

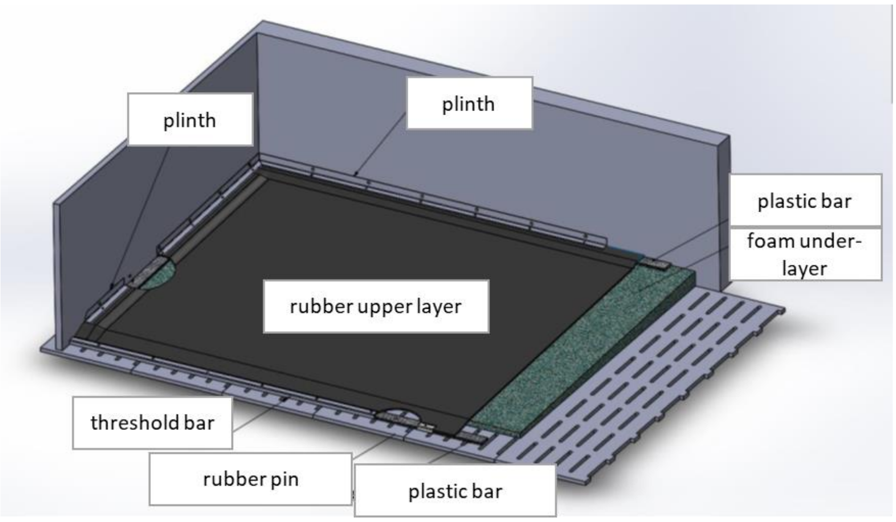

b)

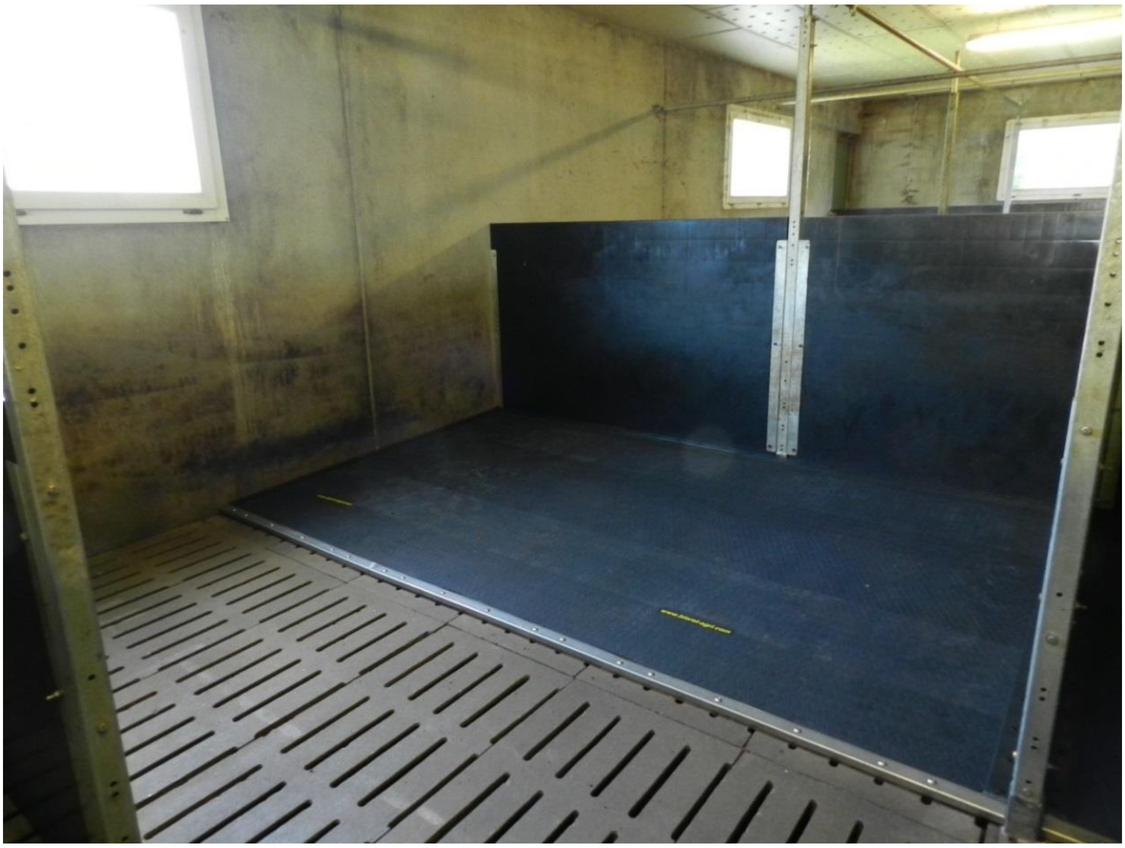

Conception of the rubber mat a) schematic representation of mat's conception, b) mat placed in a lying box
