# Supplementary material for: Do Rubber Floor Mats Prevent Lameness in Gestating Sows Housed in Large Groups? A Field Experiment on Three Commercial Farms in France
Source: Animals (Basel). 2021 Nov 5;11(11):3160. doi: 10.3390/ani11113160 (PMC8614336; doi:10.3390/ani11113160)
Supplement: Supplementary file 1 [file animals-11-03160-s001.zip › Supplementary_material_S2_v5.pdf]

Do Rubber Floor Mats Prevent Lameness in Gestating Sows Housed in Large Groups? A Field Experiment on Three Commercial Farms in France

Results of the GLM models

Table S1: Results of mixed ordinal regression model for lameness

	Lameness		
Predictor	*Odds ratio*	*CI*	*p*
No lameness | mild lameness[1]	5.05	0.67 – 38.27	0.12
Mild lameness| severe lameness[1]	44.23	5.77 – 339.32	**<0.001**
Rubber mat	0.50	0.13 – 2.01	0.33
Control	1		
Middle	2.13	1.60 – 2.84	**<0.001**
End	2.51	1.88 – 3.34	**<0.001**
Beginning	1		
Cycle 2	1.06	0.83 – 1.35	0.65
Cycle 1	1		
Parity rank = 1	0.80	0.53 – 1.21	0.30
Parity rank >5	0.74	0.43 – 1.26	0.26
Parity rank [2-5]	1		
**Random Effects**			
τ_{00} [2] $_{sow(pen:farm)}$	1.19		
$\tau_{00\ pen:farm}$	1.17		
$\tau_{00\ farm}$	2.37		
ICC [3]	0.59		
N_{sow}	495		
N_{pen}	10		
N_{farm}	3		
Observations	2361		
Marginal R^2 / Conditional R^2	0.035 / 0.604		

[1]Intercept
[2]Standard errors of the random effects
[3]Intra-class correlation

Table S2: Results of the mixed logistic regression models for bursitis and leg injuries

	**Bursitis***			**Leg injuries**		
Predictor	*Odds ratio*	*CI*	*p*	*Odds ratio*	*CI*	*p*
(Intercept)	0.03	0.00 – 7.58	0.210	0.05	0.01 – 0.31	**0.001**
Rubber mat				1.37	0.16 – 11.70	0.775
Control				1		
Rubber mat at Te	**0.29**	0.10 – 0.90	**0.032**			
Rubber mat at Tm	0.72	0.24 – 2.15	0.552			
Rubber mat at Tb	0.82	0.28 – 2.47	0.729			
Control at Te	0.82	0.54 – 1.24	0.355			
Control at Tm	0.72	0.48 – 1.09	0.119			
Control at Tb	1					
End (Te)				0.55	0.38 – 0.80	**0.002**
Beginning (Tb)				1		
Cycle 2	0.72	0.55 – 0.94	**0.014**	1.04	0.72 – 1.51	0.816
Cycle 1	1			1		
Parity rank = 1	0.39	0.22 – 0.68	**0.001**	0.52	0.26 – 1.03	0.062
Parity rank >5	1.09	0.63 – 1.87	0.767	0.82	0.52 – 13.0	0.401
Parity rank [2–5]	1			1		
**Random Effects**						
τ_{00} [1] $_{sow(pen:farm)}$	1.47			0.15		
$\tau_{00\ pen:farm}$	0.38			3.15		
$\tau_{00\ farm}$	19.19			0.30		
ICC[2]	0.86			0.52		
N sows	495			576		
N pens	10			10		
N farms	3			3		
Observations	2361			1650		
Marginal R^2 / Conditional R^2	0.013 / 0.866			0.026 / 0.535		

[1]Standard errors of the random effects
[2]Intra-class correlation

*The model includes a significant Treatment*Visit interaction

Table S3: Results of the mixed ordinal regression models for claw growth defects and claw erosion

	Claw defects			Erosion		
Predictor	*Odds ratio*	*CI*	*p*	*Odds ratio*	*CI*	*p*
Absence|mild[1]	0.79	0.19 – 3.30	0.742	0.06	0.03 – 0.14	**<0.001**
Mild|Severe[1]	14.08	3.31 – 59.84	**<0.001**	4.11	1.86 – 9.09	**<0.001**
Rubber mat	0.92	0.67 – 1.25	0.588	0.65	0.24 – 1.81	0.413
Control	1			1		
End (Te)	1.26	1.01 – 1.57	**0.037**	1.67	1.34 – 2.09	**<0.001**
Beginning (Tb)	1			1		
Cycle 1	1.58	1.26 – 1.98	**<0.001**	1.00	0.80 – 1.25	0.968
Cycle 2	1			1		
Parity rank = 1	0.11	0.07 – 0.18	**<0.001**	0.51	0.35 – 0.75	**0.001**
Parity rank >5	2.41	1.67 – 3.50	**<0.001**	1.33	0.96 – 1.83	0.086
Parity rank [2–5]	1			1		
**Random Effects**						
τ_{00} [2] $_{sow(pen:farm)}$	1.70			0.87		
$\tau_{00\ pen:farm}$	0.28			0.27		
$\tau_{00\ farm}$	1.53			0.51		
ICC[3]	0.49			0.33		
N sows	576			576		
N pens	10			10		
N farms	3			3		
Observations	1650			1650		
Marginal R^2 / Conditional R^2	0.207 / 0.541			0.051 / 0.354		

[1]Intercept

[2]Standard errors of the random effects

[3]Intra-class correlation

Table S4: Results of the GLM models for body condition measures

	Scratches			Cleanliness*			Thin			Fat		
Predictor	*Odds ratio*	*CI*	*p*	*Odds ratio*	*CI*	*p*	*Odds ratio*	*CI*	*p*	*Odds ratio*	*CI*	*p*
(Intercept)	0.13	0.04 – 0.40	**<0.001**				0.01	0.01 – 0.04	**<0.001**	0.12	0.02 – 0.97	**0.046**
(Intercept)												
Clean to dirty				4.55	3.04 – 6.81	**<0.001**						
Dirty to very dirty				43.07	27.63 – 67.13	**<0.001**						
Clean				1								
Rubber Mat	0.74	0.19 – 2.87	0.659				0.52	0.26 – 1.06	0.072	1.25	0.47 – 3.33	0.652
Control	1						1			1		
Middle (Tm)	7.77	5.76 – 10.49	**<0.001**				1.54	0.88 – 2.68	0.132	0.85	0.63 – 1.15	0.289
End (Te)	5.17	3.85 – 6.93	**<0.001**				1.54	0.88 – 2.69	0.133	1.09	0.81 – 1.46	0.577
Beginning (Tb)	1											
Cycle 2	0.52	0.42 – 0.66	**<0.001**	1.03	0.85 – 1.25	0.745	0.43	0.26 – 0.72	**0.001**	0.83	0.64 – 1.09	0.178
Cycle 1	1			1			1			1		
Parity rank = 1	1.10	0.77 – 1.57	0.608	1.66	1.25 – 2.20	**<0.001**	0.35	0.12 – 1.00	0.051	0.65	0.38 – 1.09	0.099
Parity rank >5	0.74	0.50 – 1.11	0.151	0.70	0.49 – 0.99	**0.047**	0.84	0.32 – 2.22	0.724	1.29	0.70 – 2.38	0.408
Parity rank [2–5]	1			1			1			1		
Rubber mat at Te				29.27	16.96 – 50.49	**<0.001**						
Rubber mat at Tm				5.14	3.03 – 8.71	**<0.001**						
Rubber mat at Tb				0.57	0.32 – 1.01	0.055						
Control at Te				6.62	4.78 – 9.17	**<0.001**						
Control at Tm				1.12	0.79 – 1.58	0.534						
Control at Tb				1								
**Random Effects**												
τ_{00} [1] $_{sow(pen:farm)}$	0.60			0.39			4.69			2.34		
$\tau_{00\ pen:farm}$	1.15			0.10			0.04			0.52		
$\tau_{00\ farm}$	0.23			0.00			0.00			2.94		
ICC[2]	0.38						0.59			0.64		

N sows	495	495	495	495
N pens	10	10	10	10
N farms	3	3	3	3
Observations	2361	2360	2353	2353
Marginal R^2 / Conditional R^2	0.149 / 0.469	0.364 / NA	0.054 / 0.612	0.007 / 0.641

[1]Standard errors of the random effects

[2]Intra-class correlation

*Mixed ordinal regression model including a significant Treatment*Visit interaction

Table S5: Results of the GLM models for reproductive performance traits

	Number of piglets born			Number of piglets born alive			Number of weaned piglets		
Predictors	LSM[1]	SE[2]	p	LSM	SE	p	LSM	SE	p
(Intercept)	16.1	0.7	**<0.001**	15.6	0.5	**<0.001**	12.8	0.4	**<0.001**
Rubber mat	15.0	0.6	0.28	14.1	0.4	0.20	12.2	0.3	0.34
Control	15.6	0.6		14.7	0.4		12.4	0.3	
Cycle 1	15.3	0.6	0.87	14.6	0.4	0.292	12.6 a	0.4	**<0.001**
Cycle 2	15.4	0.4		14.3	0.3		12.0 b	0.3	
Parity rank = 1	14.6	0.6 a	**0.01**	14.1a	0.4	**0.001**	12.8 a	0.4	**<0.001**
Parity rank [2–5]	15.8	0.6 b		15.2b	0.4		12.4 a	0.3	
Parity rank >5	15.6	0.5 ab		14.0a	0.4		11.7 b	0.3	
Random Effects									
τ_{00} [4] $_{pen:farm}$	0.50			0.34			0.04		
$\tau_{00\ farm}$	0.19			0.00			0.21		
ICC[5]	0.03			0.03			0.06		
N pen	10			10			10		
N farm	3			3			3		
Observations	801			786			791		
Marginal R^2 / Conditional R^2	0.014 / 0.046			0.029 / 0.054			0.046 / 0.099		

[1]Least square mean
[2]Standard error
[3]Means with different subscripts are different at p <0.05 (Tukey adjustment)
[4]Standard errors of random effects
[5]Intra-class correlation
